# Structural and biochemical characterization of a trapped coenzyme A adduct of *Caenorhabditis elegans* glucosamine-6-phosphate *N*-acetyltransferase 1

**DOI:** 10.1107/S0907444912019592

**Published:** 2012-07-17

**Authors:** Helge C. Dorfmueller, Wenxia Fang, Francesco V. Rao, David E. Blair, Helen Attrill, Daan M. F. van Aalten

**Affiliations:** a Division of Molecular Microbiology, College of Life Sciences, University of Dundee, Dundee DD1 5EH, Scotland

**Keywords:** carbohydrates, glycobiology, *Caenorhabditis elegans*, glucosamine-6-phosphate *N*-acetyltransferase, coenzyme A adduct, mechanism

## Abstract

Glucosamine-6-phosphate *N*-acetyltransferase 1 (GNA1) produces GlcNAc-6-phosphate from GlcN-6-phosphate and acetyl coenzyme A. Early mercury-labelling experiments implicated a conserved cysteine in the reaction mechanism, whereas recent structural data appear to support a mechanism in which this cysteine plays no role. Here, two crystal structures of *Caenorhabditis elegans* GNA1 are reported, revealing an unusual covalent complex between this cysteine and the coenzyme A product. Mass-spectrometric and reduction studies showed that this inactive covalent complex can be reactivated through reduction, yet mutagenesis of the cysteine supports a previously reported bi-bi mechanism. The data unify the apparently contradictory earlier reports on the role of a cysteine in the GNA1 active site.

## Abstract 2

Glucosamine-6-phosphate *N*-acetyltransferase is an essential enzyme of the eukaryotic UDP-GlcNAc biosynthetic pathway. A crystal structure at 1.55 Å resolution revealed a highly unusual covalent product complex and biochemical studies investigated the function of a fully conserved active-site cysteine.

## 1. Introduction

UDP-GlcNAc is an essential metabolite in both prokaryotes and eukaryotes. In eukaryotes, UDP-GlcNAc serves as a donor substrate for the synthesis of N- and O-linked glycans and polymeric carbohydrates such as chitin and hyaluronan (Hanover *et al.*, 1987 ▶; Hart *et al.*, 1989 ▶; Glaser & Brown, 1957 ▶; Yoshida *et al.*, 2000 ▶). It also serves as the substrate for the synthesis of the glycosylphosphatidylinositol (GPI) anchors of cell-wall proteins (Watanabe *et al.*, 1996 ▶). In eukaryotes, UDP-GlcNAc is synthesized from fructose 6-phosphate (Fru-6P) and glutamine in four enzymatic steps known as the UDP-GlcNAc biosynthetic pathway (Milewski *et al.*, 2006 ▶). The first step, the conversion of Fru-6P and glutamine to glucosamine 6-­phosphate (GlcN-6P), is carried out by the bifunctional enzyme glutamine:fructose-6-phosphate amidotransferase (GFA1). GlcN-6P is then acetylated by the enzyme glucos­amine-6-phosphate *N*-acetyltransferase 1 (GNA1), followed by conversion to GlcN-1P by a phosphomutase (AGM1) and finally generation of UDP-GlcNAc by the action of a pyrophosphorylase (Lagorce *et al.*, 2002 ▶; Milewski *et al.*, 2006 ▶).

**Figure 1:**
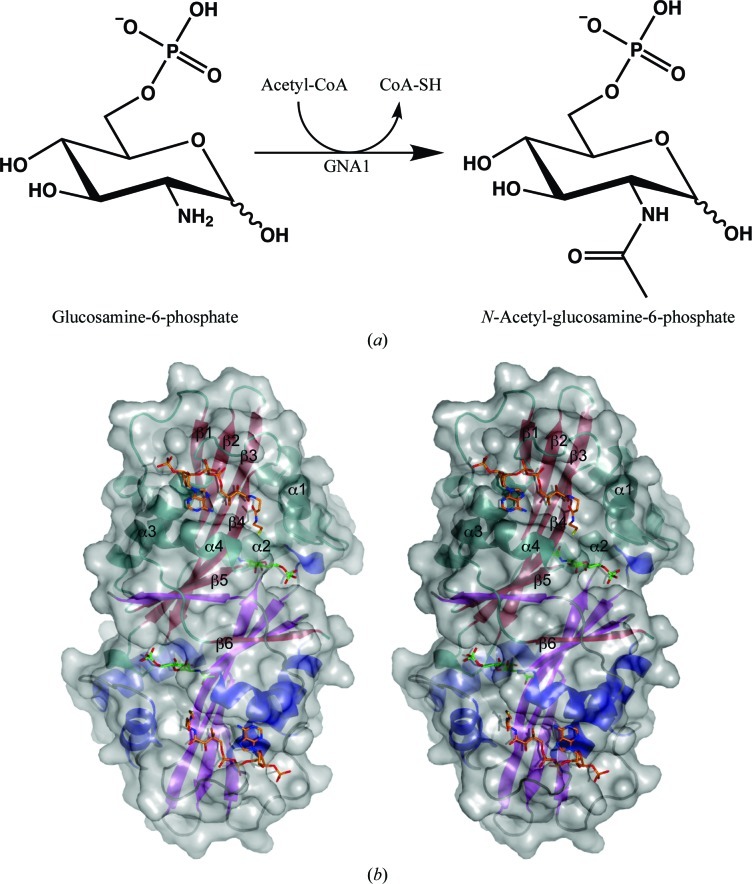
(*a*) Glucosamine-6-phosphate *N*-acetyltransferase (GNA1) catalyses the *N*-acetylation of d-­glucosamine 6-phosphate (GlcN-6P) using acetyl-CoA as an acetyl donor. The product GlcNAc-6P is an intermediate in the biosynthesis of UDP-GlcNAc. (*b*) Stereo figure showing a structural overview (in grey surface and ribbon representation) of the intertwined noncrystallographic *Ce*GNA1 dimer. Secondary-structure elements of one subunit are labelled and coloured green (helices) and red (β-strands). The products CoA (orange) and GlcNAc-6P (green) are shown in stick representation binding in the corresponding subsites at the dimer interface where the β6 strand exchanges.

GNA1 is a member of the Gcn5-related *N*-acetyltransferase (GNAT) superfamily (Peneff *et al.*, 2001 ▶). The enzymes of this family catalyse the transfer of an acetyl group from the donor acetyl coenzyme A to acceptor amino groups on proteins or sugars (Dyda *et al.*, 2000 ▶; Fig. 1 ▶ *a*). Structural analyses have revealed a conserved GNAT core that is involved in binding of acetyl-CoA (Vetting, de Carvalho, Roderick *et al.*, 2005 ▶). Members of the GNAT family include enzymes as diverse as aminoglycoside *N*-acetyltransferases (AgNATs), serotonin *N*-acetyl­transferases (SNATs) and histone acetyltransferases (HATs; Vetting, de Carvalho, Yu *et al.*, 2005 ▶; Shaw *et al.*, 1993 ▶; De Angelis *et al.*, 1998 ▶; Brownell *et al.*, 1996 ▶). GNA1 has been purified and characterized from several organisms, including human (Zwierz *et al.*, 1976 ▶; Hopwood *et al.*, 1983 ▶; Vessal & Jaberi-Pour, 1998 ▶), pig (Porowski *et al.*, 1990 ▶), rat (Oikawa & Akamatsu, 1985 ▶; Oikawa *et al.*, 1986 ▶), mouse (Boehmelt, Fialka *et al.*, 2000 ▶; Boehmelt, Wakeham *et al.*, 2000 ▶), mosquito (Kato *et al.*, 2005 ▶), *Neurospora crassa* (Pattabiraman & Bachhawat, 1962 ▶; Davidson *et al.*, 1957 ▶), *Candida albicans* (Mio *et al.*, 2000 ▶) and *Trypanosoma brucei* (Mariño *et al.*, 2011 ▶). The most extensively studied GNA1 is from the yeast *Saccharomyces cerevisiae* (*Sc*GNA1; Mio *et al.*, 1999 ▶, 2000 ▶; Peneff *et al.*, 2001 ▶), for which functional, enzymatic and structural data are available. *Sc*GNA1 is essential for growth (Mio *et al.*, 1999 ▶) and is thought to be the only amino-sugar acetyltransferase in yeast, given that the only acetylated amino sugar detected in yeast to date is GlcNAc; both GalNAc and ManNAc are thought to be absent from yeast cells (Mio *et al.*, 1999 ▶). Studies with *C. albicans* GNA1 (*Ca*GNA1) null mutants have shown reduced virulence in a mouse model and consequently the enzyme has been suggested as a possible antifungal drug target (Mio *et al.*, 2000 ▶). However, inhibitors of GNA1 have not yet been reported and given the existence of a functional orthologue in humans it is currently not clear whether specific inhibitors can be developed.

Two different reaction mechanisms for members of the GNAT family have been suggested in the literature. The first mechanism involves transfer of the acetyl group from acetyl-CoA by a single-step mechanism with the deprotonated amine on the substrate as the nucleophile (ternary-complex mechanism) on the acetyl C atom (Dyda *et al.*, 2000 ▶; Lau *et al.*, 2000 ▶; Peneff *et al.*, 2001 ▶). The second mechanism involves two steps (substituted-enzyme mechanism) *via* a covalent acetyl-enzyme intermediate through a conserved cysteine (Corfield *et al.*, 1984 ▶; Vessal & Jaberi-Pour, 1998 ▶). The data obtained by two enzymological studies of rat liver GNA1 were consistent with the substituted-enzyme mechanism (Corfield *et al.*, 1984 ▶). However, the crystal structures of *Sc*GNA1 in complex with substrates and products have recently been reported and are not in agreement with such a mechanism (Peneff *et al.*, 2001 ▶). The structures showed that there was no conserved cysteine in the immediate vicinity of the substrate, and combined with mutagenesis studies (Mio *et al.*, 2000 ▶) showing the involvement of a conserved tyrosine a ternary-complex mechanism was therefore proposed (Peneff *et al.*, 2001 ▶). Strikingly, a conserved active-site cysteine with unknown function is observed within 6.5 Å of the acetyl-CoA. The structure of *Aspergillus fumigatus* GNA1 (*Af*GNA1) has recently been solved and the authors used the pseudo-substrate glucose 6-phosphate (Glc-6P) as a probe to trap in *Af*GNA1 crystals the first GNAT (pseudo-)Michaelis complex (Hurtado-Guerrero *et al.*, 2007 ▶). These data have provided direct evidence for the nucleophilic attack of the substrate amine and have given insight into the protonation of the thiolate leaving group.

To allow the rational design of mechanism-based inhibitors, a detailed description of the role of key residues in the active site is required. To date, only a few inhibitors have been reported for members of the GNAT family. For instance, bisubstrate analogues have been described to be potent inhibitors of arylalkylamine *N*-acetyl­transferase (SNAT; Khalil *et al.*, 1999 ▶; Zheng & Cole, 2003 ▶), gentamicin acetyltransferase (Williams & Northrop, 1979 ▶) and histone acetyltransferase (Sagar *et al.*, 2004 ▶). More recently, a series of aminoglycoside–CoA bisubstrate analogues have been synthesized (Gao *et al.*, 2005 ▶, 2006 ▶). To date, no specific inhibitors are known for the GNA1 enzymes, although inhibition by nonspecific compounds has been reported. *N. crassa* GNA1 was inhibited completely by 1 m*M* *p*-chloromercuribenzoate (Davidson *et al.*, 1957 ▶). Partial inhibition of GNA1 could also be achieved with *p*-hydroxymercuribenzoate (Davidson *et al.*, 1957 ▶; Pattabiraman & Bachhawat, 1962 ▶; Vessal & Hassid, 1973 ▶; Oikawa & Akamatsu, 1985 ▶; Corfield *et al.*, 1984 ▶; Vessal & Jaberi-Pour, 1998 ▶). These inhibition studies have been taken to suggest that cysteines must be present near the active site (Vessal & Hassid, 1973 ▶) or that cysteines are required for enzyme activity (Oikawa & Akamatsu, 1985 ▶). The reducing agent β-mercaptoethanol has been shown to protect rat liver GNA1 isoforms from inactivation (Oikawa & Akamatsu, 1985 ▶). Inhibition of sheep GNA1 and *Phaseolus aureus* GNA1 was reversible in the presence of l-cysteine (Pattabiraman & Bachhawat, 1962 ▶; Vessal & Hassid, 1973 ▶). Similarly, thiol-reactive compounds did not inhibit human GNA1 when dithiothreitol (DTT) was included in the enzymatic reaction (Vessal & Jaberi-Pour, 1998 ▶).

To gain further insight into the possible involvement of a cysteine residue in the active site, we have investigated the structure of *Caenorhabditis elegans* GNA1 solved by Hg-SAD and refined to 1.55 Å resolution and the kinetic properties of a mutant of a key conserved cysteine near the catalytic machinery. The data show that the cysteine is not essential for catalysis, supporting the earlier structural studies of the yeast enzyme. Strikingly, however, we observed that a stable covalent complex between CoA and the enzyme can be formed, involving a disulfide bond to the conserved cysteine, as revealed by the 1.55 Å resolution crystal structure. This modification inactivates the enzyme, which can be reversed with reducing agents. These data suggest that this conserved active-site cysteine could regulate flux through the hexos­amine-biosynthetic pathway in a redox-dependent manner and we investigate this potential regulatory mechanism using a yeast *gna1* mutant.

## 2. Methods

### 2.1. Cloning, expression and purification

A construct encoding full-length *Ce*GNA1 was PCR-amplified (forward primer, 5′-GAGAGGATTCATGT­CTC­ACATTTTTGATGCGTC-3′; reverse primer, 5′-GAA­GGAATTCTTAGAAGCGCTGAGTCATAAAATT-3′) from *C. elegans* cosmid B0024 DNA (Sanger Institute, Cam­bridge­shire, England). The PCR product was cloned into the pGEX-6P-1-plasmid using *Bam*HI and *Eco*RI sites. Site-directed mutagenesis (C141S) was performed using the QuikChange method (Stratagene) with the forward primer 5′-­CAAAATTTCTCTCGAGTCTGTTCCTGAACTTCTCCC-3′ and the reverse primer 5′-GGGAGAAGTTCAG­GAACAGACTCGAGAGAAATTTTG-3′ using standard protocols. Both DNA constructs were verified by DNA sequencing (The Sequencing Service, College of Life Sciences, University of Dundee, Scotland). *Ce*GNA1-pGEX-6P-1 constructs were transformed into *Escherichia coli* BL21 (DE3) pLysS cells. Cells were grown overnight at 310 K in Luria–Bertani (LB) medium containing 50 µg ml^−1^ ampicillin. 10 ml of the overnight culture was used to inoculate 1 l LB medium. The bacteria were grown until an OD_600_ of 0.6 was reached, induced by the addition of 0.5 m*M* isopropyl β-d-1-thio­galactopyranoside and cultured for 4 h at 310 K. Cells were harvested by centrifugation for 25 min at 3500 rev min^−1^ (277 K). The pellet from 1 l culture was washed with 40 ml fresh medium and centrifuged for 25 min at 3500 rev min^−1^ (277 K). The cells were flash-cooled in liquid nitrogen, thawed at 310 K and resuspended in 25 ml ice-cold buffer *A* (25 m*M* Tris–HCl pH 7.5, 250 m*M* NaCl, 2 m*M* EDTA) with 1 mg ml^−1^ lysozyme (Sigma), 1 mg ml^−1^ DNAse and half a protease-inhibitor tablet (Roche) including 1 m*M* DTT. The cell pellets were sonicated on ice for 4 × 30 s with a 1 cm diameter sonicator probe (Sanyo Soniprep 150). The fractions were centrifuged for 25 min at 13 500 rev min^−1^ (Beckman Avanti-J25, JA 25.50). The supernatant was filtered through a Minisart 0.45 µm syringe filter prior to binding to prewashed glutathione Sepharose beads for 2 h (277 K). The N-terminal GST tag was removed from the fusion protein by incubating the beads with PreScission Protease (120 µg) at 277 K overnight. The supernatant of the beads and the subsequent wash were passed over a 20 ml disposable column (Bio-Rad) to remove the GST beads. The resulting filtrate was concentrated to 4 ml and loaded onto a Superdex 75 26/60 gel-filtration column pre-equilibrated in buffer *A*. Pure fractions were verified by SDS–PAGE, pooled and spin-concentrated using a 10 000 molecular-weight cutoff concentrator.

### 2.2. Crystallization, phasing and refinement

Protein (17 mg ml^−1^) was pre-incubated on ice for 1 min with a final concentration of 5 m*M* of both substrates (acetyl-CoA and GlcN-6-P) as well as 5 m*M* of the product *N*-acetyl-d-glucosamine 6-phosphate. The sitting-drop vapour-diffusion method was used to produce crystals by mixing 0.5 µl of the protein/ligand solution with an equal volume of mother liquor [0.1 *M* Tris–HCl pH 8.5, 0.2 *M* sodium acetate trihydrate and 30%(*v*/*v*) PEG 3350 in the presence of 11 m*M* BaCl_2_] at 293 K. Bar-shaped crystals (space group *P*2_1_2_1_2_1_) grew within 2 d and bipyramidal-shaped crystals (*P*6_1_) grew within 12 d. The crystals were cryoprotected using 10% ethylene glycol in the mother liquor (*P*2_1_2_1_2_1_) or 10% glycerol in the mother liquor (*P*6_1_) and were cooled in a nitrogen-gas stream at 100 K. A single-wavelength SAD experiment was carried out using an in-house generator (Rigaku MicroMax-007 rotating-anode generator equipped with an R-AXIS IV^++^ detector) using a native hexagonal crystal and a crystal soaked for 20 min in mother liquor containing 20 m*M* HgCl_2_. The soaked crystal was cryoprotected with mother liquor containing 5% PEG 400. Data were processed with the *HKL* suite (Otwinowski & Minor, 1997 ▶). Phasing, phase extension and solvent flattening were performed with *SOLVE* (Terwilliger & Berendzen, 1999 ▶), exploiting the anomalous signal of three mercury sites. The resulting 2.0 Å resolution electron-density map was partially autotraced using *ARP*/*wARP* (Perrakis *et al.*, 1999 ▶), followed by refinement with *CNS* (Brünger *et al.*, 1998 ▶) interspersed with model building with *O* (Jones *et al.*, 1991 ▶). This model was used to solve the subsequent structures *Ce*GNA1–Cys–CoA and *Ce*GNA1–CoA–GlcNAc-6P to 1.55 and 1.75 Å resolution, respectively, using data collected on BM14, ESRF, Grenoble. Ligands were included when unambiguously defined by unbiased |*F*_o_| − |*F*_c_|, ϕ_calc_ maps. Ligand topologies and coordinates were generated with *PRODRG* (Schüttelkopf & van Aalten, 2004 ▶). The full polypeptide chain was built for both structures, with the exception of Met1. However, poor electron density was observed in the following regions. In the *P*6_1_ structure chain *A* residues 16–17, 143–151 and chain *B* residues 17–20 are disordered. In addition, the adenosine moiety of the CoA molecule had poor electron density. Consequently, these regions have higher *B* factors. The side chains of Leu18, Ser41, Ser45, Ser76, Ser99, Pro143 and Glu144 in subunit *A* and of Ser41, Ser76 and Leu122 in subunit *B* did not appear to have dual conformations. In the *P*2_1_2_1_2_1_ structure residues 16–20 are slightly disordered in molecule *A* as well as the β-mercaptoethylamine (bME) moiety of the CoA molecule in subunit *B*. Two ethylene glycol molecules are bound to the backbone N and O atoms of Val89 in subunits *A* and *B* of the *P*2_1_2_1_2_1_ structure. All residues in the *P*6_1_ data set occupy allowed regions of the Ramachandran plot. One amino acid (0.3%) in the *P*2_1_2_1_2_1_ data set was built into a disallowed region. This residue (Leu18 in chain *A*) is part of the partially disordered region 16–20. All figures were produced with *PyMOL* (DeLano, 2004 ▶).

### 2.3. Enzymology and mass spectrometry

Steady-state kinetics of wild-type (WT) and C141S-mutant *Ce*GNA1 were determined using a previously described protocol (Riddles *et al.*, 1979 ▶; Gehring *et al.*, 1996 ▶) with some experimental changes. Substrates, CoA and DTNB were supplied by Sigma. All measurements were performed in triplicate. Standard reaction mixtures consisted of 3 n*M* *Ce*GNA1 or 10 n*M* C141S *Ce*GNA1 in buffer *A* [25 m*M* Tris–HCl pH 7.5, 250 m*M* NaCl, 2 m*M* EDTA, 5%(*v*/*v*) glycerol] in a total volume of 50 µl incubated at RT (293 K). The assays were initiated by adding the protein and were stopped after 3 min (WT) or 5 min (C141S mutant) using 50 µl of a solution consisting of 25 m*M* Tris–HCl pH 7.5, 250 m*M* NaCl, 2 m*M* EDTA, 6.4 *M* guanidine chloride. 50 µl of a DTNB solution (1 m*M* DTNB in 0.1% DMSO) containing 25 m*M* Tris–HCl pH 7.5, 250 m*M* NaCl and 2 m*M* EDTA was added to determine the absorbance at 412 nm using a SpectraMax 340PC (Molecular Devices). The absorbance intensity data were analyzed using nonlinear regression analysis with *GraFit* (Fig. 3*a*; Leatherbarrow, 2001 ▶) and *GraphPad Prism* (Fig. 3*b*; http://www.graphpad.com) using the default equations for first-­order reaction rates and Michaelis–Menten steady-state kinetics.

A second assay was carried out to detect the activity of the protein in the crystals in the presence of increasing concentrations of dithiothreitol (DTT). This assay was based on the detection of the second product of the reaction, *N*-acetyl-d-­glucosamine 6-phosphate, by reducing-end labelling with *p*-­dimethylaminobenzaldehyde (DMAB; Sigma; Reissig *et al.*, 1955 ▶). 15–20 crystals of space group *P*6_1_ were harvested, washed three times in the mother liquor of the crystallization condition and dissolved in 3 µl reaction buffer (25 m*M* bis-Tris-propane pH 8.0, 250 m*M* NaCl, 2 m*M* EDTA). The reaction was performed in 2 ml sealable glass vials. 10 n*M* enzyme was incubated with the indicated concentrations of DTT for 15 min and aliquots were analyzed by mass spectrometry. Both substrates (acetyl-CoA and glucosamine 6-phosphate) were then added to a final concentration of 1 m*M* and the reaction was run for 15 min at 293 K (RT). The addition of 25 µl borate solution was used to stop the reaction. After immediate vortexing, the solution was heated in a vigorously boiling water bath for 3 min. The vials were placed in cold water at approximately 283 K; 750 µl of the diluted DMAB solution was then added. After vortexing, the tubes were stored at 310 K for 15 min. An aliquot of 300 µl was transferred to a plastic 96-well plate and the absorbance was measured at 585 nm.

To establish pH–activity profiles, apparent *K*_m_ values (acetyl-CoA) and *k*_cat_ were determined by Michaelis–Menten kinetics for the pH range 6–10 in 0.5 pH-unit steps using the DTNB assay described above, with the exception that buffer *A* was made with 25 m*M* bis-Tris-propane. The acetyl-CoA concentration was varied (0, 50, 100, 200, 300, 400, 500 and 600 µ*M*) in the presence of an excess of GlcN-6P (1.25 m*M*). Data were fitted *versus* the pH using the double-bell equation in the program *GraFit* (Leatherbarrow, 2001 ▶).

### 2.4. Yeast strains

*S. cerevisiae* strain GNA1/gna1::KanMX6 (BY4743; Mat a/a; his3D1/his3D1; leu2D0/leu2D0; lys2D0/LYS2; MET15/met15D0; ura3D0/ura3D0; YFL017c::kanMX4/YFL017c) was obtained from EUROSCARF and grown at 303 K on YPDA solid medium. A PCR product containing full-length *Sc*GNA1 with a GST-PreScission protease cleavage site was digested with *Hin*dIII–*Xho*I and cloned into a digested pYES2 plasmid (Invitrogen), resulting in the plasmid pYES-GST-*Sc*GNA1. Site-directed mutagenesis (C135S) of *Sc*GNA1 was performed using the QuikChange method (Stratagene) with standard protocols (pYES-GST-Cys135Ser). Plasmids were transfected into GNA1/gna1::KanMX6 strain using the electroporation method (Manivasakam & Schiestl, 1993 ▶). Dropout plates without uracil/uridine but containing 200 µg ml^−1^ G418 and 1% glucose (DOA-ura + G418 + 1% Glu) were used to select successfully transfected GNA1/gna1::KanMX6 with plasmids. The colonies were first streaked on GNA plates (5% d-­glucose, 3% Difco Nutrient Broth, 1% Difco Yeast Extract, 2% Bacto Agar) for 1–2 d active growth at 303 K. Colonies were then patched onto VB sporulation plates (0.82% sodium acetate, 0.19% KCl, 0.035% MgSO_4_, 0.12% NaCl, 1.5% agar) and incubated at 299 K to allow tetrad formation. After 3–5 d, when at least 20% of the cells looked like tetrads, tetrad dissection was performed under a microscope on DOA-ura + G418 plates containing 1% galactose and 1% raffinose (DOA-ura + G418 + 1% Gal + 1% Raf) to select for gna1::KanMX6 haploids with transfected plasmids. Genomic DNA and plasmids from dissected haploid cells were extracted. Primers AB, AkanB and AD of YFL017C (http://med.stanford.edu/sgtc/) were used to verify GNA1 deletion from the haploid strain. The primers GST-N (AAACGATGGCCCCTATACTAGG­TTA) and *Sc*GNA1-C (CTATTTTCTAATTTGCATTTC­CACG) were used to ensure the existence of pYES-GST-*Sc*GNA1 and pYES-GST-*Sc*GNA1-Cys135Ser in the haploid strain.

### 2.5. *In vivo* expression and purification of WT and C135S-mutant *Sc*GNA1

Protein expression of GST-*Sc*GNA1 and GST-*Sc*GNA1-Cys135Ser was induced by galactose following the method for the pYES expression vector (Invitrogen) with the following modifications. Briefly, cells were first cultured overnight in DOA-ura + 1% raffinose medium and then inoculated into DOA-ura + 1% galactose and DOA-ura + 1% glucose media for 26 h expression. Cells were harvested and disrupted using acid-washed glass beads and vortexing. After 10 min of centrifugation at 12 000 rev min^−1^ and 277 K, the soluble protein concentration was assayed using the Bradford method. 12% SDS–PAGE and Western blotting using GST antibody were used to verify expression under different conditions. Purification using GST beads was performed as previously described. Pure protein was sent for MALDI-TOF mass spectrometry. To study the effects of oxidative stress, a final concentration of 0.5 m*M* H_2_O_2_ was added to cells when the OD_600_ reached 0.1. Calculated H_2_O_2_ according to the actual OD_600_ and culture volume was added to the medium for a further 3 h expression. The protein was analyzed and purified as described previously.

## 3. Results

### 3.1. *Ce*GNA1 is a homodimer that adopts the GNAT fold

*Ce*GNA1 was cloned and overexpressed as a GST-fusion protein in *E. coli*. The recombinant protein was purified using glutathione-affinity and size-exclusion chromatography and was crystallized from PEG solutions. Two crystal forms, an orthorhombic and a hexagonal form, were obtained under identical conditions. The hexagonal crystal form was used for structure solution using a single-wavelength anomalous dispersion experiment with a mercury derivative, yielding experimental phases to a resolution of 2.0 Å and a good quality electron-density map that was partially automatically interpreted followed by manual building and refinement to 1.55 Å resolution, yielding the final model with statistics shown in Table 1 ▶. The orthorhombic crystal form was solved by molecular replacement and refined to 1.75 Å resolution, and represents a product complex with CoA and GlcNAc-6P that will be used to discuss the overall structure of *Ce*GNA1.

The three-dimensional structure of *Ce*GNA1 revealed a noncrystallographic homodimer (C_α_ r.m.s.d. = 0.36 Å; Fig. 1 ▶ *b*), in agreement with the elution profile from the size-exclusion column and the previously published *Sc*GNA1 (PDB entry 1i1d; C_α_ r.m.s.d. = 1.3 Å; Peneff *et al.*, 2001 ▶), *Af*GNA1 (PDB entry 2vxk; C_α_ r.m.s.d. = 1.4 Å; Hurtado-Guerrero *et al.*, 2008 ▶), human GNA1 (PDB entry 2o28; C_α_ r.m.s.d. = 1.1 Å; Wang *et al.*, 2008) and *Tb*GNA1 (PDB entry 3i3g; C_α_ r.m.s.d. = 1.6 Å; Mariño *et al.*, 2011 ▶) structures. The dimer has approximate dimensions of 70 × 36 × 38 Å. Each monomer (165 amino acids) contains the GNAT structural motifs (Supplementary Fig. 1^1^) and is similar to *Sc*GNA1 (C_α_ r.m.s.d. = 1.3 Å) as predicted from sequence alignment (31% sequence identity; Supplementary Fig. 1^1^). Each *Ce*GNA1 monomer in the dimer contains an antiparallel β-sheet composed of six β-strands, with β6-strand exchange at the dimer interface where the active sites are located. Each β-sheet is flanked by four helices from the first subunit: α1 and α2 on one site and α3 and α4 on the other side (Fig. 1 ▶ *b*). The dimer interface of *Ce*GNA1 (2822 Å^2^; Krissinel & Henrick, 2007 ▶) is in good agreement with the characterized *Sc*GNA1 (2941 Å^2^; Peneff *et al.*, 2001 ▶).

### 3.2. The *Ce*GNA1 ternary product complex reveals a conserved substrate-binding site

**Figure 2:**
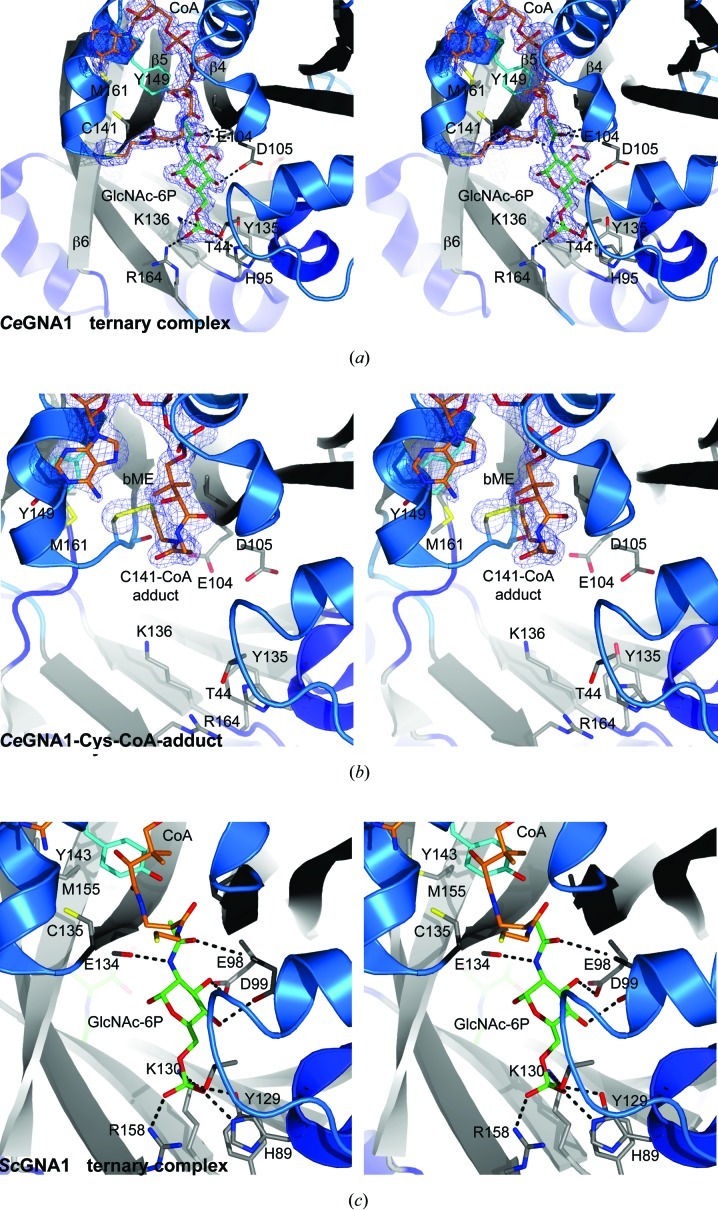
Stereo figure showing the structural dissimilarities of both obtained *Ce*GNA1 complexes in comparison to *Sc*GNA1. (*a*) Active site of one subunit of the ternary *Ce*GNA1–CoA–GlcNAc-6P complex. Active-site residues and the products are labelled and displayed as sticks. OMIT 1.75 Å resolution |*F*_o_| − |*F*_c_|, ϕ_calc_ electron density (dark blue) is shown for both products. Tyr149 points into the active site, while Cys141 is flipped out. (*b*) *Ce*GNA1–Cys–CoA adduct showing the alternate conformations of Tyr149, Met161 and Cys141 compared with (*a*). OMIT 1.55 Å resolution |*F*_o_| − |*F*_c_|, ϕ_calc_ electron density is shown for the product CoA and the disulfide bond to Cys141. Tyr149 lines the wall of the active site, while Cys141 points into the active site and is covalently linked to CoA. (*c*) *Sc*GNA1 in the ternary complex (adopted from Peneff *et al.*, 2001 ▶; PDB entry 1i1d). Tyr143, Cys135 and Met155 are in similar conformations as in the ternary *Ce*GNA1 complex. The product GlcNAc-6P is hydrogen bonded to equivalent residues.

The active sites of *Ce*GNA1 in the orthorhombic crystal form are occupied by the products CoA and GlcNAc-6-phosphate, both with well defined electron density except for the β-mercaptoethyl­amine (bME) moiety of one of the CoA molecules in the asymmetric unit (Fig. 2 ▶ *a*), generating a ternary complex. Given that acetyl-CoA, GlcN-6P and GlcNAc-6P were added to the crystallization mother liquor, the enzyme must have turned over at least some of the available acetyl-CoA, generating free CoA. The active site consists of two substrate-binding subsites: the acceptor (GlcN-6P) subsite and the acetyl-CoA subsite. The GlcN-6P binding site is located at the dimer interface. Residues from both subunits contribute to the binding of the 6-­phosphate group. Two of the five residues located at the base of this cleft are positively charged (Lys136 and Arg164) and interact with the negatively charged phosphate group (Fig. 2 ▶ *a*). These five residues are completely conserved between *Ce*GNA1 and *Sc*GNA1 (Supplementary Fig. 1^1^). Several hydrogen bonds are established between the GlcN sugar and conserved residues: the Glu140 backbone carbonyl forms hydrogen bonds to the acetamido N atom, while Glu104 (β4) and Asp105 (β5) form hydrogen bonds to the 3-hydroxyl and 4-­hydroxyl groups, respectively (Fig. 2 ▶ *a*). The backbone N atoms of Asp105 and Val106 form hydrogen bonds to the carbonyl acetyl group (Fig. 2 ▶ *a*). The tyrosine that has previously been proposed to participate in catalysis in *Sc*GNA1 (Tyr143) adopts a similar conformation in *Ce*GNA1 (Tyr149) (Figs. 2 ▶ *a* and 2 ▶ *c*). The conserved active-site residues Cys141 (*Ce*GNA1) and Met161 (*Ce*GNA1) are also in similar conformations compared with *Sc*GNA1 (Figs. 2 ▶ *a* and 2 ▶ *c*). The thiol group of Cys141 lies 6.0 Å away from the superimposed acetyl-CoA acetyl group and is therefore 0.5 Å further away than the corresponding Cys135 in the *Sc*GNA1 structure. The CoA molecule is bound in a largely hydrophobic cleft where β4 and β5 diverge. The adenosine moiety sits on top of the α3 helix (Fig. 2 ▶ *a*). The β-­mercapto­ethylamine moiety points out of the active site, away from the GlcN-6P product (Fig. 2 ▶ *a*). Similar to what is observed in the *Sc*GNA1 ternary product complex (PDB entry 1i1d), one of the two CoA molecules is partially disordered in its bME moiety (PDB entry 4ag7).

### 3.3. A conserved active-site cysteine forms a covalent CoA adduct

**Figure 3:**
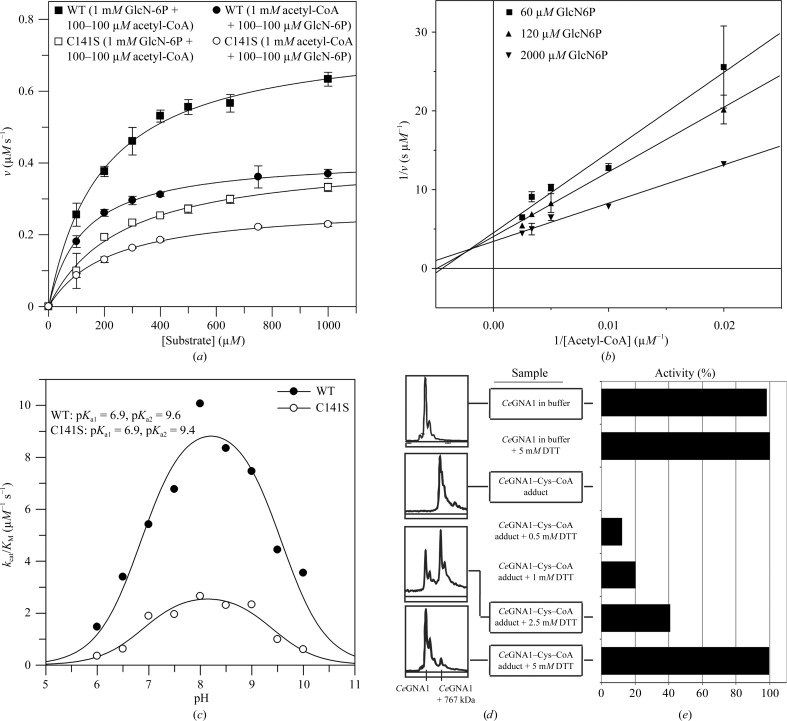
(*a*) Apparent *K*_m_ of WT and C141S *Ce*GNA1 for both substrates. The velocity (µ*M* s^−1^) is shown as a function of the concentration of the second substrate. The WT enzyme has approximately twofold higher affinity for both substrates than the active-site mutant C141S *Ce*GNA1. The catalytic efficiency of the mutant has dropped to around 20% (Table 2 ▶). However, the covalent adduct shows no detectable signal above the background (Table 2 ▶). (*b*) Double-reciprocal plots of initial rates of formation of CoA catalysed by *Ce*GNA1. In this plot, the concentration of acetyl-CoA is varied from 50 to 500 µ*M* at several fixed concentrations of GlcN-6P. (*c*) pH optima of WT and C141S *Ce*GNA1. To obtain the bell-shaped curves the nonlinear fit was calculated with *GraFit*. WT and C141S *Ce*GNA1 have the same pH optimum at pH 8.2. (*d*) Mass spectrometry supports the covalent bond between the active-site Cys141 and the product CoA in the crystal structure (space group *P*6_1_). 15 min incubation (at 293 K) with 5 m*M* DTT was sufficient to release the covalently bonded CoA, corresponding to the mass of one released CoA molecule (767 Da). (*e*) Activity of the *Ce*GNA1–Cys–CoA adduct dimer compared with *Ce*GNA1 in solution after incubation with various concentrations of DTT as indicated. Incubation of 10 n*M* *Ce*GNA1–Cys–CoA without DTT does not give any detectable activity above the background. Reactivation of 98.2% can be achieved with 5 m*M* DTT for 15 min at 293 K.

In addition to the crystal form containing a product complex described above, further crystals of a different form (hexagonal; space group *P*6_1_) appeared under the same conditions. When these crystals were investigated by diffraction, structure solution and subsequent refinement (Table 1 ▶), a number of interesting features were observed (Fig. 2 ▶ *b*). The active site is only occupied by one product, the CoA molecule, but the CoA thiol has formed a covalent complex with the enzyme through a disulfide with a conserved cysteine that has undergone a dramatic conformational change (Figs. 2 ▶ *a* and 2 ▶ *b*). In both the *Ce*GNA1 and *Sc*GNA1 product complexes the thiol of this cysteine is completely buried, facing away from the active site (Figs. 2 ▶ *a* and 2 ▶ *c*). In the structure of the covalent CoA complex the cysteine has shifted (maximum atom shifts of 4.0 Å for the side chain and 2.0 Å for the backbone) and rotated (124° around χ_1_) from its buried position into an exposed position on the wall of the active site, positioning it at a distance of 2.0 Å from the CoA thiol (Fig. 2 ▶). Well defined electron density is present for the Cys141–CoA disulfide (Fig. 2 ▶ *b*). Further significant conformational changes have also taken place. Tyr149, the residue that is thought to stabilize the negative charge developed on the CoA thiol in the proposed reaction mechanism (Peneff *et al.*, 2001 ▶), has rotated away from the active site (130° around χ_1_). Furthermore, Met161, which forms the pocket for the buried cysteine in the product complex (Fig. 2 ▶ *a*), has changed conformation (123° around χ_1_, 45° around χ_2_). To further confirm the presence of the covalent enzyme–CoA adduct, the protein from the crystals was investigated by MALDI-TOF mass spectrometry (Fig. 3 ▶ *a*). A control protein sample taken immediately after purification had a mean mass of 18 876 ± 36 Da, whereas the protein from the dissolved crystals showed a mean mass of 19 654 ± 21 Da. The calculated difference mass of 777 Da is compatible with the theoretical mass of CoA (768 Da). Thus, mass spectrometry shows the presence of the enzyme–CoA adduct, in agreement with the crystallographic data. The crystallographic and mass-spectrometric data show that in contrast to what has been suggested for the *Sc*GNA1 structure, it is possible for Cys141, which is fully conserved in the entire GNA1 family (Supplementary Fig. 1 ▶ 1), to approach the catalytic centre. At first sight, this revives the previously published hypothesis that the conserved cysteine is involved in the reaction mechanism (Corfield *et al.*, 1984 ▶; Vessal & Jaberi-Pour, 1998 ▶).

### 3.4. The conserved Cys141 is not directly involved in the reaction mechanism

A C141S mutant was produced in order to investigate the involvement of the cysteine thiol in the reaction mechanism and was studied by steady-state kinetics (Fig. 3 ▶ *a*). The wild-type enzyme gives kinetic parameters (Table 2 ▶) compatible with previous studies (Mio *et al.*, 2000 ▶; Kato *et al.*, 2005 ▶; Jiang *et al.*, 2005 ▶). Only modest changes in the Michaelis constant (twofold higher) or in the turnover number (fivefold lower) were observed for the C141S mutant (Table 2 ▶; Fig. 3 ▶ *a*). This suggests that the cysteine is not directly involved in catalysis as the nucleophile in a substituted-enzyme mechanism.

To further confirm this, double-reciprocal plots of steady-state kinetics of the wild-type enzyme were investigated (Fig. 3 ▶ *b*). These data show that the apparent *K*_m_ of one substrate is affected by increasing concentrations of the other substrate (*i.e.* intersecting, rather than parallel, lines), again suggesting a ternary-complex mechanism for the enzyme-catalyzed reaction rather than a substituted-enzyme mechanism involving the conserved cysteine (Cleland, 1967 ▶). The ternary-complex mechanism was identified as a single-step mechanism with the deprotonated amine on the substrate acting as the nucleophile on the acetyl C atom of acetyl-CoA (Hurtado-Guerrero *et al.*, 2007 ▶). In this mechanism, the conserved Tyr149 of *Ce*GNA1 is not required for catalysis; however, it stabilizes the thiolate leaving group (Hurtado-Guerrero *et al.*, 2007 ▶).

A reaction mechanism that would involve a covalent Cys141–acetyl intermediate (*i.e.* a substituted-enzyme mechanism) would involve deprotonation of the thiol group to generate the nucleophile, assisted by a basic residue in the active site, and this deprotonation event would be effected in the C141S mutant with varying pH, *i.e.* a sigmoidal pH profile with one p*K*_a_ would be expected.

The pH-dependent activity of wild-type and C141S-mutant *Ce*GNA1 was also investigated in order to further probe the role of the conserved Cys141. The wild-type and C141S-mutant proteins yielded similar bell-shaped pH–activity profiles (Fig. 3 ▶ *c*). The p*K*_a_ values and pH optima calculated from these data are virtually indistinguishable (Fig. 3 ▶ *c*). A pH optimum of 8.2 is similar to those found in previous studies of GNA1 homologues, which generally give pH optima in the alkaline range (pH 7.4–9.7; Pattabiraman & Bachhawat, 1962 ▶; Giddings & Cantino, 1974 ▶; Oikawa & Akamatsu, 1985 ▶; Porowski *et al.*, 1990 ▶; Vessal & Jaberi-Pour, 1998 ▶). However, mutation of the cysteine to serine still yields a bell-shaped pH curve, leading to an identical pH optimum (Fig. 3 ▶ *c*).

Although this provides further support for a ternary-complex mechanism in addition to the analyses of steady-state kinetics described above, it appears to be incongruent with the structural data, which suggest that the cysteine is able to undergo a dramatic conformational change that would place it in proximity to the catalytic centre and form a covalent complex with the CoA product.

### 3.5. Reduction of the Cys141–CoA adduct leads to reactivation

In addition to the kinetic studies performed on the wild-type enzyme and the C141S mutant, the *Ce*GNA1–CoA adduct as obtained from dissolved crystals was also studied. This form of the enzyme did not show any detectable activity (Table 2 ▶). This is not surprising as the structural data show that CoA is covalently linked to Cys141, permanently occupying the acetyl-CoA binding site. It was subsequently investigated whether this form of the protein could be reactivated under reducing conditions. Reduction studies with increasing amounts of DTT showed full restoration of activity to the level observed for the recombinant protein immediately after purification (Fig. 3 ▶ *e*). The reduction of the *Ce*GNA1–CoA adduct was further investigated by mass spectrometry, revealing that increasing concentrations of DTT were able to release CoA, shifting the mass of the enzyme back to that of the purified protein prior to crystallization (Fig. 3 ▶ *d*). It thus appears that under the nonreducing conditions used for crystallization the product of the acetyltransferase reaction, CoA, is able to induce a dramatic conformational change in the active site, generating a catalytically inactive form of the enzyme through the formation of a disulfide with Cys141 that is conserved throughout the GNA1 family. Under reducing conditions this inactive form can be reactivated.

### 3.6. Oxidizing conditions do not induce the cysteine–CoA adduct in yeast GNA1 **in vivo**

To investigate whether the stable covalent complex between the conserved active-site cysteine (Cys141) in *Ce*GNA1 and the product CoA exists *in vivo*, we decided to use *S. cerevisiae* as a model system to study this adduct. The *S. cerevisiae* GNA1 homologue has been structurally characterized previously (Peneff *et al.*, 2001 ▶) and shows a conserved active-site Cys135 equivalent to Cys141 in *C. elegans* GNA1. Plasmids containing *Sc*GNA1 and the C135S mutant under control of the pGAL1 promoter were transfected into heterozygous diploid *Sc*GNA1 (YFL017C) strain. After sporulation and dissection, *Sc*GNA1-deleted haploid strains containing plasmids were selected on DOA-ura + G418 + Gal + Raf plates. PCR verification using appropriate primers showed that strains without *Sc*GNA1 but complemented by plasmids were obtained (data not shown). GST-fused wild-type *Sc*GNA1 or the C135S mutant were highly expressed from pYES-GST-*Sc*GNA1 or pYES-GST-Cys135Ser under induction by galactose and the proteins were purified for biochemical studies. Purified recombinant *Sc*GNA1 and C135S-mutant proteins were analysed by MALDI-TOF mass spectrometry (Supplementary Figs. 2*a* and 2*b* 1) and no molecular-weight shift was detected between the wild type and the mutant. As the CoA sulfhydryl group can only react with the cysteine sulfhydryl group under oxidative conditions, we investigated whether supplemention of the cells with 0.5 m*M* H_2_O_2_ for 3 h after 24 h expression would inactivate the enzyme by forming a covalent Cys135–CoA adduct; again, no evidence for the covalent CoA adduct was found (Supplementary Figs. 2*c* and 2*d* 1). Furthermore, the purified protein was supplemented with the reaction product CoA *in vitro*; MALDI-TOF analysis did not reveal any molecular-weight shift for any of these reaction conditions (Supplementary Figs. 2*e* and 2*f* 1). These data indicate that the conserved cysteine residue is not required for *Sc*GNA1 catalytic activity in the cellular context and is not involved in oxidative stress-dependent regulation of UDP-GlcNAc biosynthesis. These data are in agreement with kinetic data obtained using the purified enzymes, which show that oxidative conditions (0.5 m*M* H_2_O_2_) do not inhibit wild-type and C135S-mutant *Sc*GNA1 activity *in vitro* (100 and 96% relative activity, respectively; data not shown).

## 4. Discussion

The structural data described here have revealed the presence of an unusual covalent adduct between a fully conserved cysteine (Cys141) in the *Ce*GNA1 active site and the product CoA. Recently, the structure of the GNAT superfamily member ribosomal protein L12 (RimL) has been determined, revealing a similar covalent complex between the active-site Cys134 and the product CoA that could also be detected in solution (Vetting, de Carvalho, Roderick *et al.*, 2005 ▶). However, in contrast to *Ce*GNA1 this cysteine is only partially conserved in RimL orthologues and does not occupy an equivalent position to Cys141 in *Ce*GNA1. The redox sensitivity of the RimL disulfide was not investigated.

The *Ce*GNA1 structural data reported here suggest possible involvement of this cysteine in catalysis, which is compatible with earlier data showing that GNA1 enzymes can be inactivated by thiol-reactive compounds (Davidson *et al.*, 1957 ▶; Pattabiraman & Bachhawat, 1962 ▶; Vessal & Hassid, 1973 ▶; Oikawa & Akamatsu, 1985 ▶; Corfield *et al.*, 1984 ▶; Vessal & Jaberi-Pour, 1998 ▶). This apparently contradicted earlier structural work with the yeast GNA1 orthologue which showed that the equivalent Cys135 in *Sc*GNA1 is not involved in the catalytic mechanism as it is positioned 5.5 Å away from the acetyl group (Peneff *et al.*, 2001 ▶). This was also observed in the structures of *A. fumigatus* and human GNA1 (Hurtado-Guerrero *et al.*, 2008 ▶; Wang *et al.*, 2008 ▶). However, we demonstrate here that this conserved cysteine can undergo significant conformational/positional changes that result in it pointing into the active site. In this alternate conformation the active-site cysteine could potentially play a role in transferring the acetyl group from acetyl-CoA onto the sugar *via* an acetyl-cysteine intermediate. The discovery of this hitherto unknown alternative conformation of Cys141 (*Ce*GNA1) protruding into the active site could therefore suggest that catalysis could proceed *via* a substituted-enzyme mechanism as proposed for the rat liver enzyme (Corfield *et al.*, 1984 ▶; Vessal & Jaberi-Pour, 1998 ▶) and serotonin acetyltransferase (Watanabe *et al.*, 1992 ▶).

We addressed this question by mutational analysis of the *Ce*GNA1 cysteine. Apparent Michaelis–Menten kinetics for the wild-type and C141S-mutant *Ce*GNA1 for both substrates revealed minor differences between the wild type and the mutant. Furthermore, the wild-type and mutant pH optima were indistinguishable and were found to be in the alkaline range (pH 8.2), in agreement with what has been reported for other members of the GNA family (Wolf *et al.*, 1998 ▶; Boehmelt, Fialka *et al.*, 2000 ▶; Mio *et al.*, 2000 ▶; Kato *et al.*, 2005 ▶). The similar pH optima and calculated p*K*_a_ values for enzymatic activity of the wild-type and mutant *Ce*GNA1 as well as the bell-shaped pH–activity profile further support the conclusion that the acetylation does not involve an acetyl-enzyme intermediate *via* Cys141. Nevertheless, Cys141 is conserved in GNA1 from organisms ranging from *C. elegans* to human (Supplementary Fig. 1 ▶) and our structures show that Cys141 can rotate into the active site and form a disulfide with the CoA product.

Reduction experiments of the crystallized protein showed that this disulfide bridge can be reduced, leading to reversible recovery of activity associated with loss of covalently linked CoA as shown by mass spectrometry. Further experiments addressed the question of whether the Cys–CoA adduct has a function *in vivo*.

We have investigated yeast GNA1 under physiological and oxidative conditions. Neither of the experiments revealed a covalently modified enzyme which loses *N*-acetyl-d-glucos­amine 6-phosphate synthesis activity. Therefore, we conclude that covalent modification of the active-site cysteine and the product CoA is favoured by conditions during crystallization. However, the observation that this conserved cysteine residue can rotate into the active site and is approachable by the sulfhydryl group of the product CoA is interesting from a mechanistic point of view and raises the question why this reactive cysteine was conserved during the evolution of the enzyme.

## Supplementary Material

PDB reference: GNA1, coenzyme A adduct, 4ag7

PDB reference: ternary complex with coenzyme A and GlcNAc, 4ag9

Supplementary material file. DOI: 10.1107/S0907444912019592/cb5008sup1.pdf

## Figures and Tables

**Table 1:** Details of data collection and structure refinement Values in parentheses are for the highest resolution shell. All measured data were included in structure refinement.

	*Ce*GNA1 + HgCl_2_	*Ce*GNA1–Cys–CoA	*Ce*GNA1–GlcNAc-6P–CoA
Space group	*P*6_1_	*P*6_1_	*P*2_1_2_1_2_1_
Unit cell-parameters (Å)	*a* = *b* = 85.85, *c* = 76.98	*a* = *b* = 86.07, *c* = 77.29	*a* = 48.87, *b* = 53.35, *c* = 135.10
Resolution range (Å)	20.00–2.00	15.0–1.55 (1.61–1.55)	15.0–1.75 (1.81–1.75)
No. of observed reflections		502744	387318
No. of unique reflections		46024	34593
Multiplicity	17.2 (8.0)	4.3 (4.0)	3.6 (3.1)
〈*I*/σ(*I*)〉	38.5 (3.1)	35.7 (3.9)	28.1 (3.5)
Completeness (%)	99.6 (97.6)	97.9 (83.7)	97.3 (89.5)
*R*_merge_	0.049 (0.548)	0.054 (0.275)	0.048 (0.259)
No. of protein residues	—	328	328
No. of water molecules	—	352	411
*R*/*R*_free_	—	0.178/0.214	0.190/0.232
R.m.s.d. from ideal geometry			
Bonds (Å)	—	0.02	0.02
Angles (°)	—	2.45	2.06
〈*B*〉 (Å^2^)			
Wilson	—	26.4	24.6
Protein	—	27.4	28.9
Ligand	—	38.7	39.6
Water	—	39.6	39.3

**Table 2:** Kinetic data for wild-type and C141S *Ce*GNA1 The apparent *K*_m_ and *k*_cat_ values for acetyl-CoA and GlcN-6P and the catalytic efficiency were determined from the amounts of CoA produced. n.d., no detectable activity.

	*K*_m_ (µ*M*)	*k*_cat_ (s^−1^)	*k*_cat_/*K*_m_ (s^−1^ µ*M* ^−1^)
GlcN-6P (acetyl-CoA constant)			
WT	125 ± 9	139	0.6
C141S	216 ± 10	28	0.1
Cys–CoA	n.d.	n.d.	n.d.
Acetyl-CoA (GlcN-6P constant)			
WT	192 ± 16	252	1.3
C141S	261 ± 33	48	0.2
Cys–CoA	n.d.	n.d.	n.d.

## Footnotes

1 Supplementary material has been deposited in the IUCr electronic archive (Reference: CB5008). Services for accessing this material are described at the back of the journal.
