# Supplementary material for: Structural and biochemical characterization of a trapped coenzyme A adduct of Caenorhabditis elegans glucosamine-6-phosphate N-acetyltransferase 1
Source: Acta Crystallogr D Biol Crystallogr. 2012 Jul 17;68(Pt 8):1019–29. doi: 10.1107/S0907444912019592 (PMC3413214; doi:10.1107/S0907444912019592)
Supplement: Supplementary file 1 [file d-68-01019-sup1.pdf]

Supplementary Material

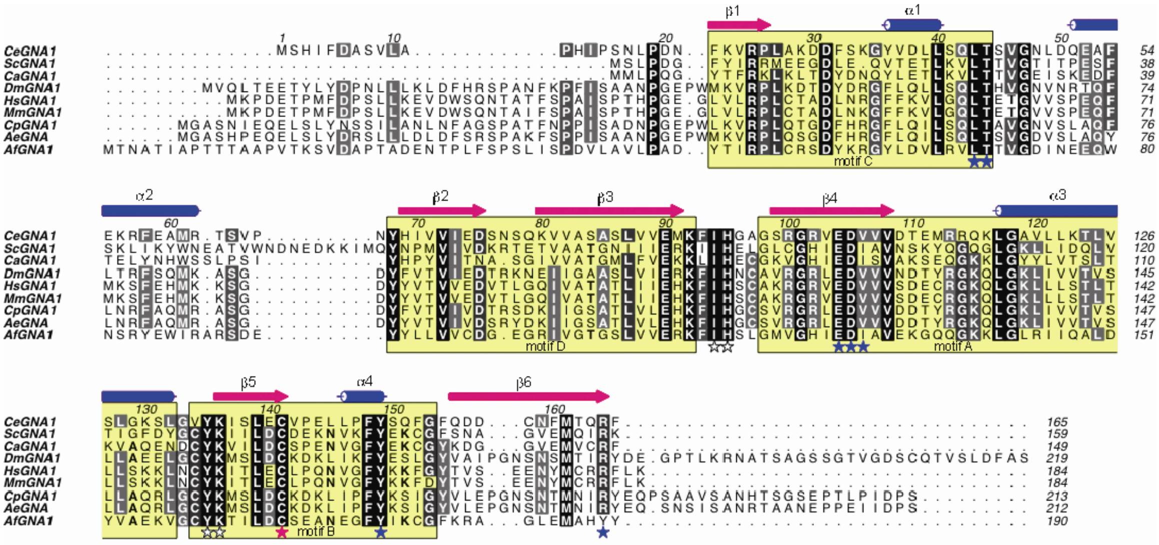

Supplementary Figure 1. Sequence alignment of GNA1 homologues generated with ALINE [Bond and Schuttelkopf, 2009]. Secondary structure elements are shown in the colour scheme used in panel A. Conserved and similar residues are highlighted with black and grey backgrounds, respectively. Active site residues involved in GlcN-6P binding are indicated with blue (subunit 1) and white stars (subunit 2). The conserved active site cysteine is identified with a pink star.

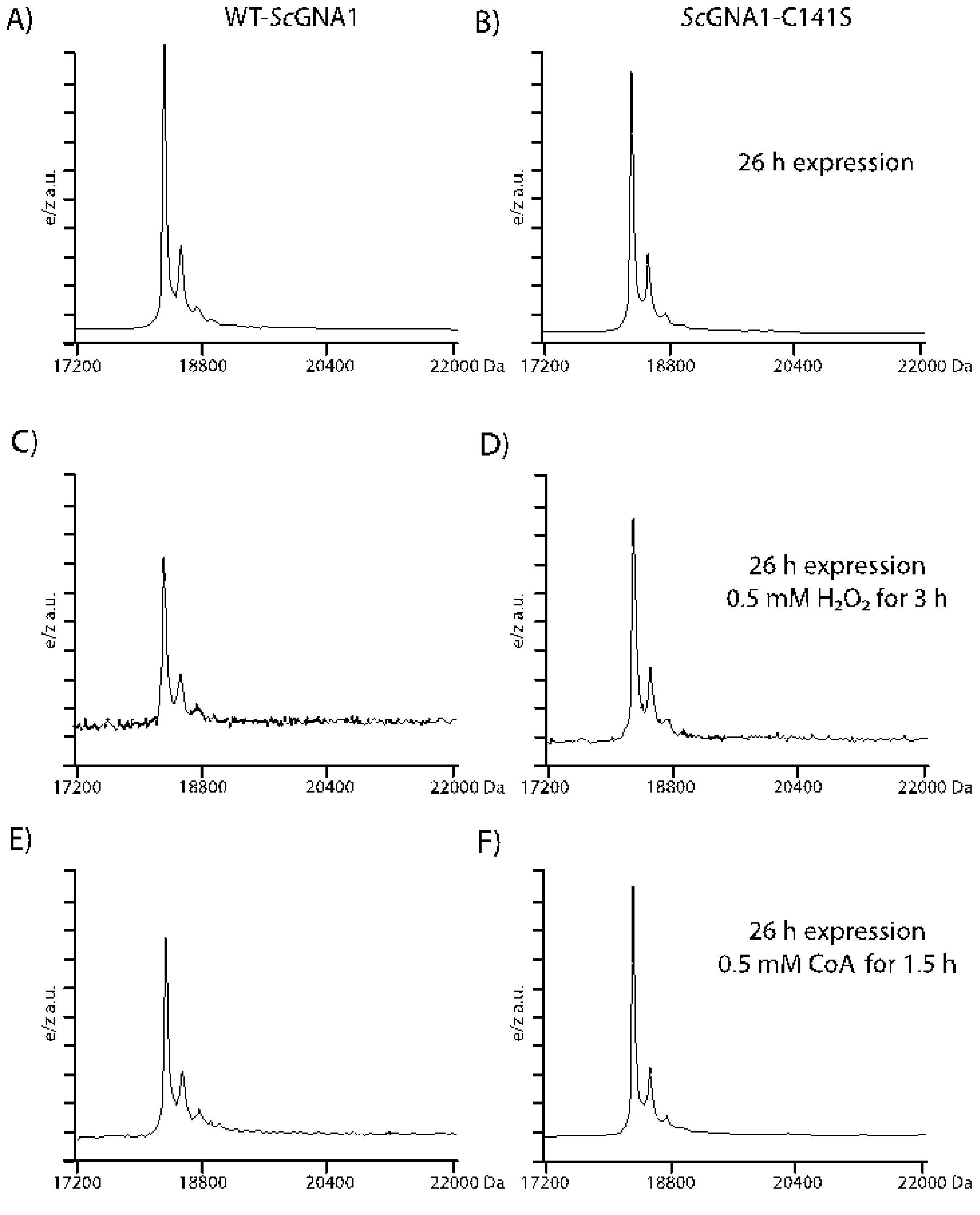

Supplementary Figure 2. Mass-spectrometry data of GST-*Sc*GNA1 and GST-*Sc*GNA1-Cys135-Ser purified from yeast under different conditions: WT and mutant protein after 26 h expression (A,B). The proteins in presence of 0.5 mM H_2O_2 (C,D) and of 0.5 mM CoA-SH (E,F). do not show a mass shift of plus 767.5 Da as observed for the crystallised *Ce*GNA1 protein (Fig. 3D).
